# The incidence, and spatial trends of cholera in Sabah over 15 years: Repeated outbreaks in coastal areas

**DOI:** 10.1371/journal.pgph.0002861

**Published:** 2024-01-30

**Authors:** Marilyn Charlene Montini Maluda, Emilia Johnson, Fredie Robinson, Muhammad Jikal, Siat Yee Fong, Mohammad Jeffree Saffree, Kimberly M. Fornace, Kamruddin Ahmed

**Affiliations:** 1 Department of Public Health Medicine, Faculty of Medicine and Health Sciences, Universiti Malaysia Sabah, Kota Kinabalu, Sabah, Malaysia; 2 Sabah State Health Department, Ministry of Health Malaysia, Kota Kinabalu, Sabah, Malaysia; 3 School of Biodiversity, One Health and Veterinary Medicine, University of Glasgow, Glasgow, United Kingdom; 4 Borneo Medical and Health Research Centre, Faculty of Medicine and Health Sciences, Universiti Malaysia Sabah, Kota Kinabalu, Sabah, Malaysia; 5 Department of Biomedical Sciences, Faculty of Medicine and Health Sciences, Universiti Malaysia Sabah, Kota Kinabalu, Sabah, Malaysia; 6 Faculty of Infectious and Tropical Diseases and Centre for Climate Change and Planetary Health, London School of Hygiene and Tropical Medicine, London, United Kingdom; 7 Saw Swee Hock School of Public Health, National University of Singapore and National University Health System, Singapore, Singapore; 8 Department of Pathology and Microbiology, Faculty of Medicine and Health Sciences, Universiti Malaysia Sabah, Kota Kinabalu, Sabah, Malaysia; 9 Research Center for Global and Local Infectious Diseases, Oita University, Oita, Japan; Monash University, AUSTRALIA

## Abstract

*Vibrio cholerae* remains a notable public health challenge across Malaysia. Although the Malaysian state of Sabah is considered a cholera-affected area, gaps remain in understanding the epidemiological trends and spatial distribution of outbreaks. Therefore, to determine longitudinal and spatial trends in cholera cases data were obtained from the Sabah State Health Department for all notified cases of cholera between 2005–2020. A cholera outbreak is defined as one or more confirmed cases in a single locality with the evidence of local transmission. All records were geolocated to village level. Satellite-derived data and generalised linearized models were used to assess potential risk factors, including population density, elevation, and distance to the sea. Spatiotemporal clustering of reported cholera cases and zones of increased cholera risk were evaluated using the tau statistic (τ) at 550m, 5km and 10km distances. Over a 15-year period between 2005–2020, 2865 cholera cases were recorded in Sabah, with a mean incidence rate of 5.6 cases per 100,000 (95% CI: 3.4–7.9). From 2015–2020, 705 symptomatic cases and 727 asymptomatic cases were reported. Symptomatic cases primarily occurred in local Malaysian populations (62.6%, 441/705) and in children and adolescents under 15-years old (49.4%, 348/705). On average, cases were reported in areas with low population density (19.45 persons/km^2^), low elevations (19.45m) and near coastal areas. Spatiotemporal clustering of cholera cases was identified up to 3.5km, with increased village-level cholera risk within 500m and 5 days of initial case presentation to a health facility (Risk Ratio = 9.7, 95% CI: 7.5–12.4). Cholera incidence has high spatial and temporal heterogeneity within Sabah, with some districts experiencing repeated outbreaks. Cholera cases clustered across space and time, with village-level risk of cholera highest within 5 days and within close proximity to primary case villages, suggesting local transmission.

## Introduction

Cholera remains a public health threat, causing an estimated 1.2–4.0 million cases and 21,000–143,000 deaths worldwide. Currently, the highest incidence rates of cholera are in the countries of Sub-Saharan Africa; however, cases are also reported across Southeast Asia **[1, 2].** Cholera is caused by the bacterium *Vibrio cholerae*, a food and water borne disease with a wide clinical spectrum that ranges from asymptomatic colonization to acute watery diarrhea resulting in severe dehydration which, if untreated, may lead to hypovolemic shock, metabolic acidosis and ultimately death **[3, 4].** Infected individuals may shed the bacteria before and after the onset of symptoms for about 1 to 2 weeks whereas asymptomatic carries may shed the organism in their stools and serve as reservoir **[5].** Cholera is an outcome of interacting socio-economic-health-environmental factors, universal access to safe drinking water and adequate sanitation, which are the main measures to prevent outbreaks **[6, 7].**

More than 200 *V*. *cholerae* serogroups have been identified; however, only O1 and O139 serogroups cause major epidemics and pandemics. The serogroup O139 emerged in the Indian subcontinent in 1992 and spread across parts of Asia until mid-2000s but eventually was superseded by O1 **[4].** Serogroup O1 is further classified into two biotypes, classical and El Tor. Cholera has been in existence for the past 200 years and the first six pandemics were caused by classical biotype of *V*. *cholerae* O1. The current ongoing seventh cholera pandemic is caused by the El Tor biotype, which appeared in Indonesia in 1961 **[4].**

Malaysia is a federal country consisting of 13 states and 3 federal territories. Cholera is a notifiable disease in Malaysia and is required to be reported under the jurisdiction of the Communicable Disease Control Division, Ministry of Health, Malaysia. In Malaysia, cholera outbreaks occur periodically and are caused mainly by *V*. *cholerae* O1 El Tor, with infections by O139 occurring sporadically **[8, 9]**. In the past, cholera outbreaks occurred in the states of Sarawak, Kelantan, and Terengganu **[8–10].** The modes of transmission most frequently reported are consumption of contaminated drinking water, contaminated cooked food, and raw or undercook seafood **[11]**. In certain areas of Sarawak cholera remains endemic due to inadequate water supply and sanitary conditions. Communities in these areas depend on contaminated rivers for water supply, especially during periods of water shortages **[12].**

Sabah is another state situated on the northern part of Borneo Island neighboring Sarawak. The state has a land area of 73,904 km^2^ and is divided into 25 districts. The climate is tropical with high humidity and year-round rainfall, with increased rainfall between November and March **[13]**. Outbreaks of cholera have been documented in Sabah since 2005. The control and prevention program for cholera is under the directive of the Sabah State Health Department. Although commercial vaccines are available for the disease, currently this is not part of the routine immunization program in the state. Despite being a cholera-affected state, there is a significant gap in our understanding of the epidemiology of the disease as published data remains sparse. Therefore, the study aimed to explore and analyze epidemiological trend of cholera outbreaks in Sabah for the last 15 years. The objectives of this study were to describe the incidence of reported cholera cases within Sabah, evaluate potential environmental risk factors, and determine the rate and changes in disease incidences over time and space. This study provides insights into the dynamics of cholera outbreaks in this state to contribute to policy decisions to prevent and control cholera.

## Methodology

### Study setting and population

A state-wide database of notified cases and asymptomatic carriers of cholera during 2005–2020 was obtained from the Sabah State Health Department, Ministry of Health Malaysia. Notified cases were detected by passive surveillance and asymptomatic carriers were detected by active surveillance. Data collection is part of the routine cholera surveillance and is reported from all district health offices throughout the state. Data on age, gender and occupation were extracted to assess populations affected by cholera. The authors had access to information that could identify individual participants during or after data collection. For all cases between 2015–2020, geographical data included the districts and villages coordinates of the identified cases.

### Ethical approval

Ethical approval was obtained from the National Medical Research Register, Ministry of Health, Malaysia (NMRR-21-804-59485). The study uses secondary data therefore, obtaining consent was not required.

### Definition of epidemiological and clinical variables

The clinical case definition for cholera was defined by the Ministry of Health, Malaysia Guidelines. Clinical cholera cases were defined as any persons with acute watery diarrhea with or without vomiting. A confirmed case was defined as any person with acute watery diarrhea with or without vomiting that is laboratory-confirmed by isolation of *V*. *cholerae* O1 or O139 by culture from stool sample. A suspected case is a case that meets the clinical case definition without laboratory confirmation **[14]**. Cholera asymptomatic carriers were individuals without signs and symptoms of cholera but *V*. *cholerae* O1 or O139 was detected by culture or PCR from stool sample.

*V*. *cholerae* were isolated from stool culture of cases and asymptomatic carriers at the district level laboratories and transferred to the Kota Kinabalu Public Health Laboratory for confirmation, biotyping, and serotyping. Cholera asymptomatic carriers were detected during mass rectal swab testing as part of routine disease control measures during an outbreak. Swabs were collected from all the residents of the locality from where a cholera cases has been confirmed. The locality is defined as the whole residential area that is registered under the district office. A cholera outbreak is defined as one or more confirmed cases in a single locality with the evidence of local transmission **[15].**

Different nationalities were defined according to their country of origin. Sea Gypsies, locally known as the ‘Palauh’, are nomads and practice a seafaring lifestyle. They reside in coastal districts; Kudat on the west coast and Semporna on the east coast of Sabah **[16, 17]**.

### Mapping cholera cases

**Fig 1:**
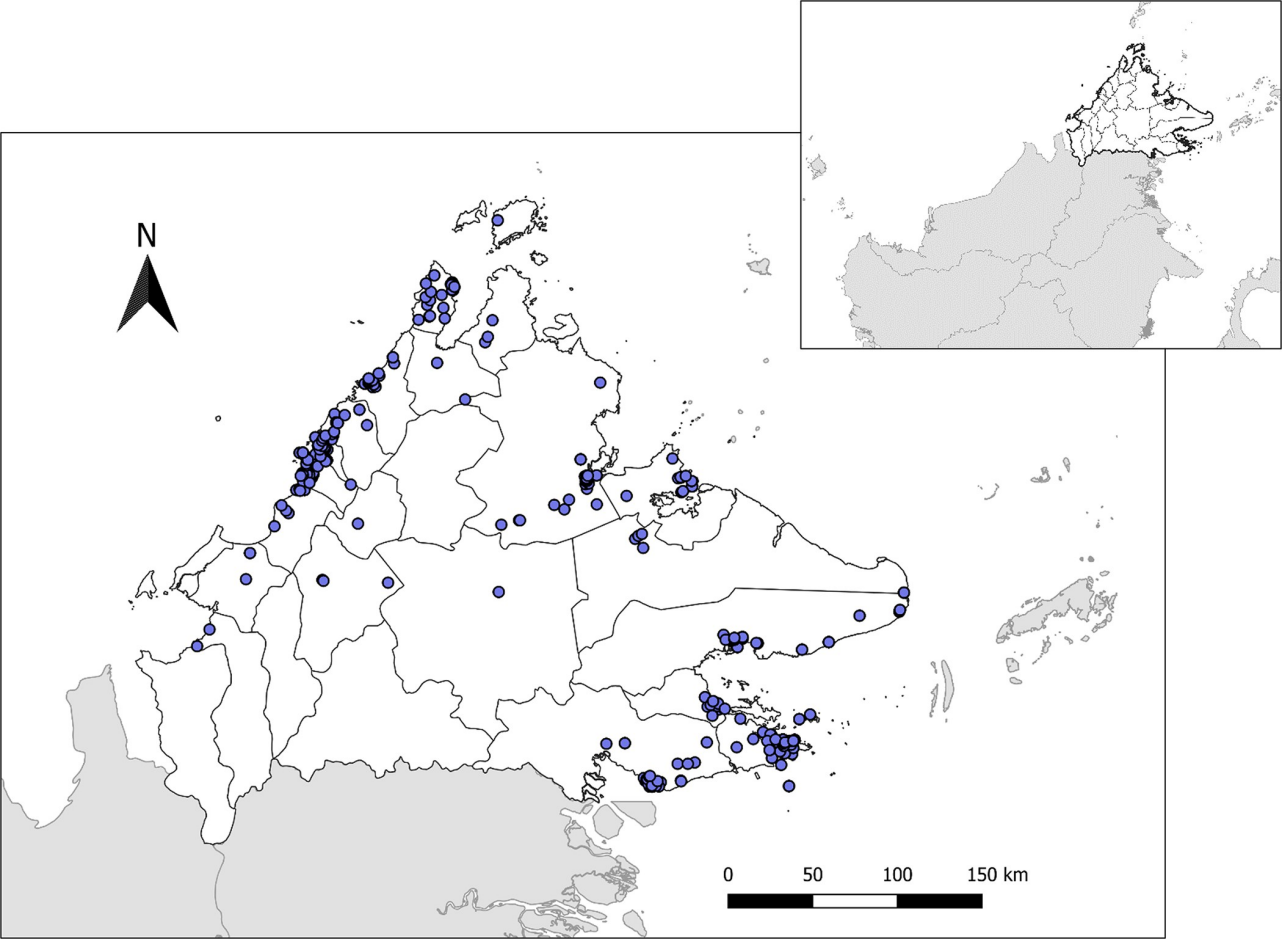
Spatial distribution of cholera cases presenting to health facilities in Sabah between 2015–2020 *(N = 705)*. Cholera cases showed a predilection for the coastal regions. The inset showing Sabah located on the northern part of Borneo Island. Base maps in were obtained from the UN OCHA Humanitarian Data Exchange platform [Malaysia - Subnational Administrative Boundaries - Humanitarian Data Exchange (humdata.org)], available for use under the following CC BY license conditions [Data Licenses - Humanitarian Data Exchange (humdata.org)].

The annual cholera incidence in a district was calculated by dividing the number of suspected and confirmed cholera cases reported each year by the population in the area. The population data was obtained from the Ministry of Health Malaysia. The mean of the annual incidence for the period was calculated for each district and expressed as per 100,000 population. Annual incidence metrics per district were merged to administrative boundaries and mapped using Quantum GIS **[18]**. Additionally, cholera cases were geolocated to village level (Fig 1). For records without household coordinates, centroids of villages were manually located using open-source remote sensing data (e.g. Open Street Maps). Potential risk factors were assembled to describe characteristics of locations with reported cases. These variables included population density **[19]** and Euclidean distance to the sea and elevation **[20]**. For all geolocated case locations, covariate values were extracted and summarized.

### Spatiotemporal clustering

Maps of cholera case locations were made using QGIS. Using R statistical software, a distance matrix was calculated to visualise the geographic range and spread of the geolocated datapoints. To assess the spatiotemporal clustering of cases, we calculated the tau statistic (τ), a global clustering statistic that estimates the relative risk (RR) of a subsequent cholera case occurring within *d* distance and within *t* days (0–5) after a suspected primary case is notified at a registered health facility, compared with the risk of a subsequent case occurring within *t* days anywhere within the study population **[21]**. For temporally linked pairs of cases, τ is defined with the following equation:

$$\hat{\tau }(d_{1},d_{2})=\frac{\hat{\theta }(d_{1},d_{2})}{\hat{\theta }(0,\infty )}$$

IDSpatialStats package in R **[22]** was used to calculate τ, exploring risk within distance bands at increments of 50m between 0m and 550m. Finer spatial increments were precluded by the broad spatial spread of the cases across Sabah, resulting in data sparsity within narrower distance bands. Tau statistic has been shown to be robust to heterogeneities in the underlying population distribution, and also robust to differences in detection rate and changes in disease incidence over space (distance to health facility) and over time (as is typical of case reporting during an outbreak) **[23]**. 95% confidence intervals around tau estimates were calculated from 1000 bootstrap replicates.

We estimated tau at distance intervals up to 550m, consistent with the maximum distance identified for cholera risk zones in rural areas **[24]**, and within a 5-day infection window (*t* = 0–5), which is approximately the serial interval within which cholera cases can be considered potentially linked. Zones of increased risk for cholera around cases were considered as extending until relative risk was no longer elevated above spatial randomness, or until 95% CIs for tau estimates cross the line of unity (RR = 1). Geolocations for the cholera cases were predominantly village centroids. Therefore, to account for the degrees of geographical uncertainty, we extended the exploratory distance up to 10 kilometres (10,000m) at intervals of 1,000m and 5 kilometres (5,000m) at intervals of 500m to investigate spatiotemporal clustering across Sabah at the village level.

## Results

### Symptomatic cholera

**Fig 2:**
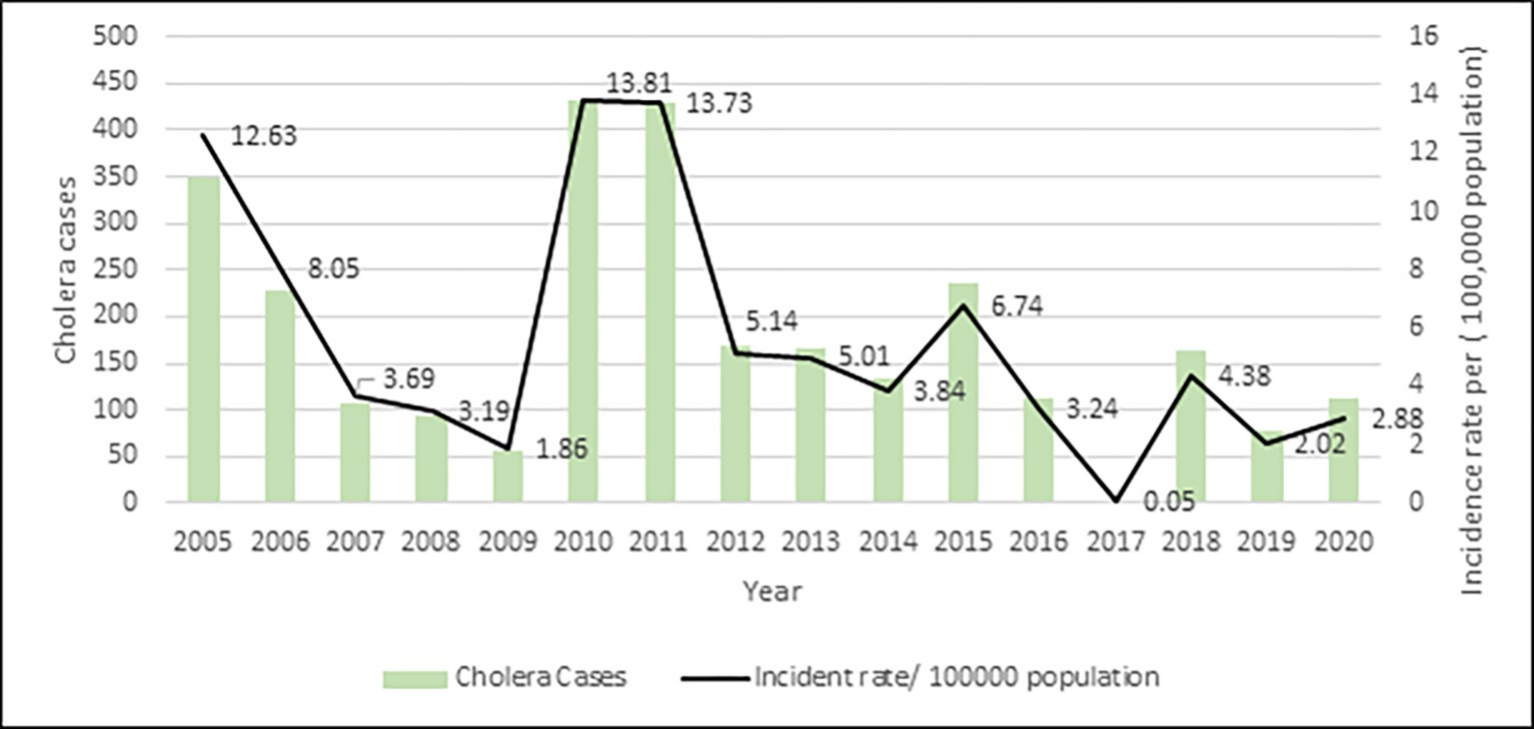
State-wide annual cholera cases and incidence rate per 100,000 populations in Sabah. The highest and lowest number of cases (431 and 2) and incidence rate (13.8 and 0.05) were detected in 2010 and 2017, respectively.

From 2005 through 2020, a total of 2,865 cholera cases were confirmed in Sabah. The annual trend of cholera (Fig 2) shows that the highest and lowest number of symptomatic cases and incidence rate were detected in 2010 (431 cases, 13.8/100,000 population) and 2017 (2 cases, 0.05/100,000 population), respectively. The mean number of symptomatic cases and the incidence rate during these 15 years were 191 and 5.6/100,000 population (95% CI: 3.4–7.9).

Data on age, gender and affected districts were available from 2015 onwards; during this period, 705 symptomatic cholera cases were reported. The majority of cholera cases were Malaysian (62.6%, 441/705 cases), followed by Filipinos (26.2%, 185/705 cases), Indonesians (5%, 35/705 cases) and 6.2% (44/705 cases) identified among the Sea Gypsies (Table 1).

**Table 1:** Distribution of symptomatic cholera cases and asymptomatic carriers (data from 2015 onwards) by nationality.

Nationality	Symptomatic Cholera cases (%)	Asymptomatic carriers (%)
**Malaysian**	441 (62.6)	403 (55.4)
**Filipino**	185 (26.2)	278 (38.2)
**Indonesian**	35 (5.0)	7 (1.0)
**Sea Gypsy**	44 (6.2)	39 (5.4)
**Total**	705 (100)	727 (100)

**Fig 3:**
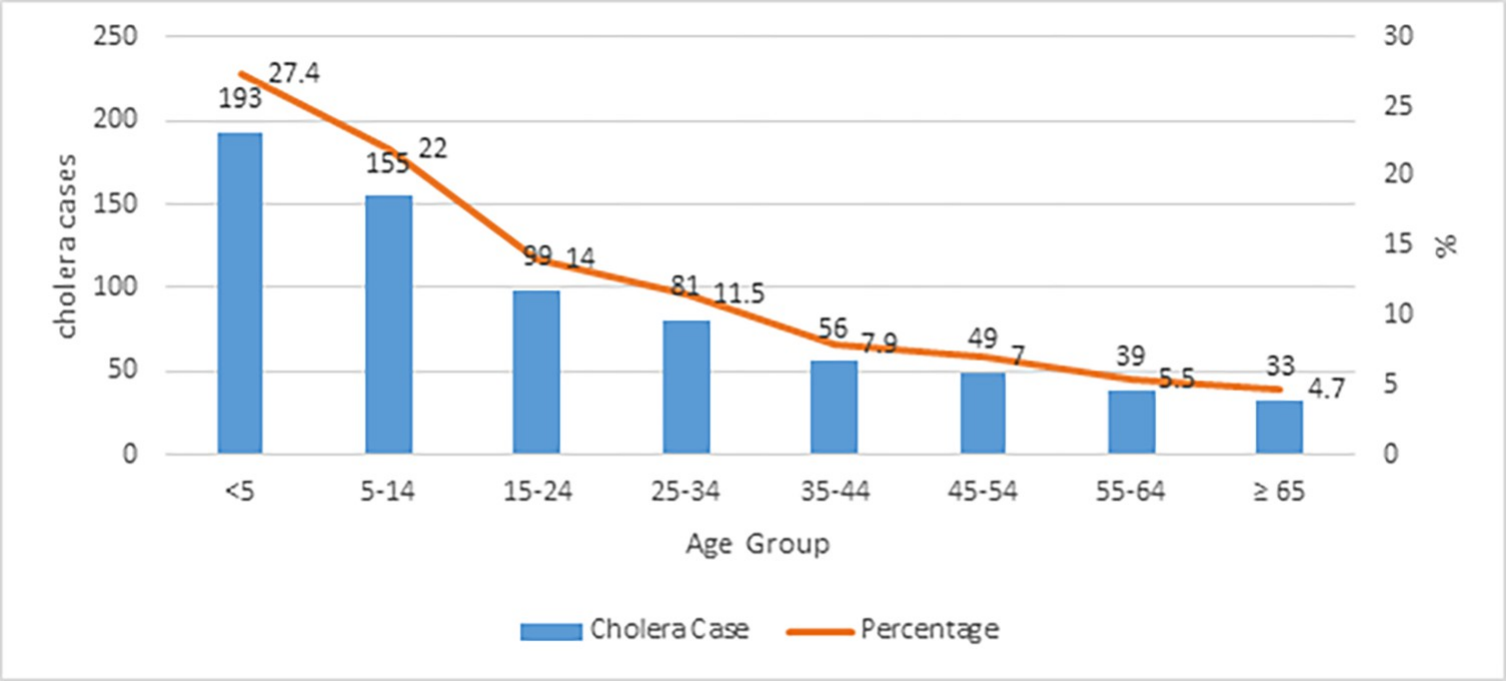
Distribution of cholera cases in Sabah by age group. Almost half of cholera cases occurred in children under 15 years of age.

Almost all *V*. *cholerae* (99.6%, 701/704) detected in symptomatic cases belonged to serogroup O1 El Tor. These strains were isolated from 359 females and 345 males with a mean age of 21.7 years (ranged 0–86 years). Almost half (49.4%, 348/705) of cholera cases were reported in individuals under 15 years, including 193 cases (27.4%, 193/705) in children under 5 years of age and 155 cases (22%, 155/705) in individuals aged 5 to 14 years (Fig 3). Individuals above 55 years old contributed to 10.2% (72/705 cases) of cases with the remaining cases reported in the age group of 15–54 years (40.4%, 285/705 cases).

**Fig 4:**
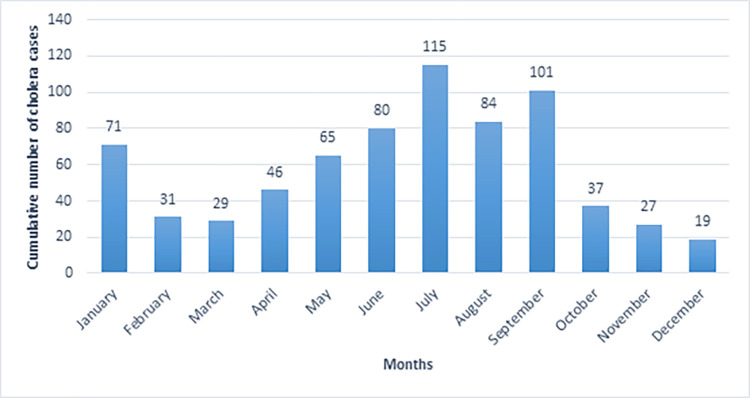
The seasonal occurrence of cholera cases in Sabah is represented by plotting the cumulative number of cholera cases for each month during 2015–2020. Cholera was occurring yearlong however, peaked in January, July, and September.

**Fig 5:**
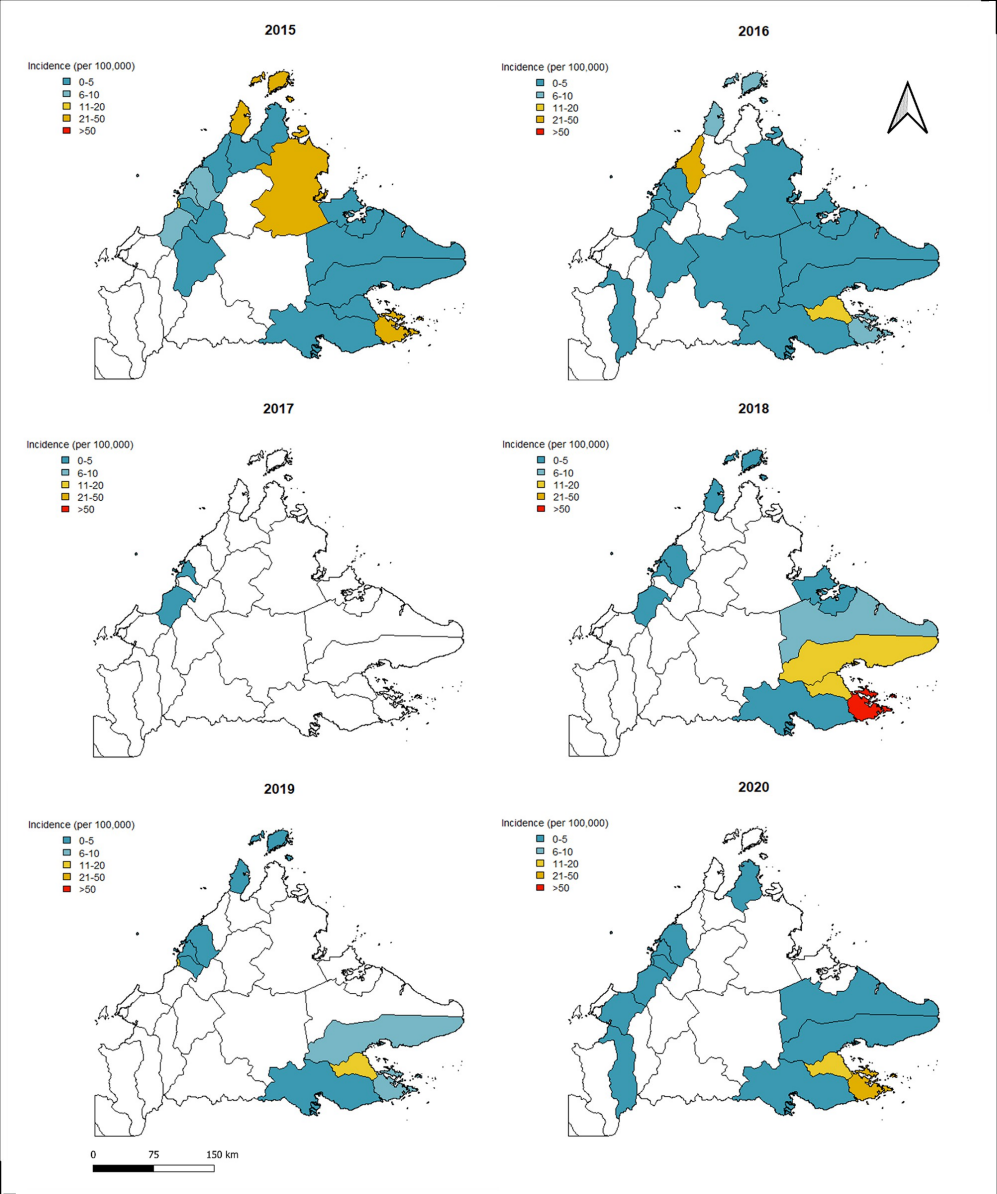
Annual cholera incidence (per 100,000 people). Shown for each district in Sabah, Malaysia, between 2015 and 2020. Blank districts indicate no case data for a given year. Base maps were obtained from the UN OCHA Humanitarian Data Exchange platform [Malaysia - Subnational Administrative Boundaries - Humanitarian Data Exchange (humdata.org)], available for use under the following CC BY license conditions [Data Licenses - Humanitarian Data Exchange (humdata.org)].

Plots of the monthly distribution of reported symptomatic cholera cases between 2015 to 2020 showed that the highest number of cholera cases occurred during June to September, peaking in July (Fig 4). The annual mean number of reported cholera cases was 118 (range: 2–235) with mean incidence rate (IR) of 3.2/100,000 population (95% CI: 0.85–5.58/100,000 population). During this five-year time period, the highest annual number of cases (235 cases) and incidence rate (6.74/100,000 population) were detected in 2015 (S1 and S2 Tables). Cholera cases were reported from all districts in Sabah except for Ranau, Tenom, Nabawan, and Kuala Penyu. The highest number of cases were reported from Beluran (55 cases), followed by Kudat (37 cases), Kota Kinabalu (37 cases), and Semporna (35 cases). The highest district-level annual incidence rate was reported from Kudat (IR: 51.8 / 100,000 population) in 2015 (S1 Table). From districts reporting cases, the lowest number of cases (2 cases) was reported in 2017 from Kota Kinabalu and Papar districts, which corresponded with the lowest mean annual incidence reported in Sabah (IR: 0.05/100,000 population). Overall in this time period, the highest mean incidence rates were reported from Semporna (20.6/100,000 population, 95% CI: 0.6–40.7) followed by Kunak (10.9/100,000 population, 95% CI: 2.7–19.1), and Kudat (10.7/100,000 population, 95% CI: 0.1–32.3) (Fig 5).

### Asymptomatic cholera

**Fig 6:**
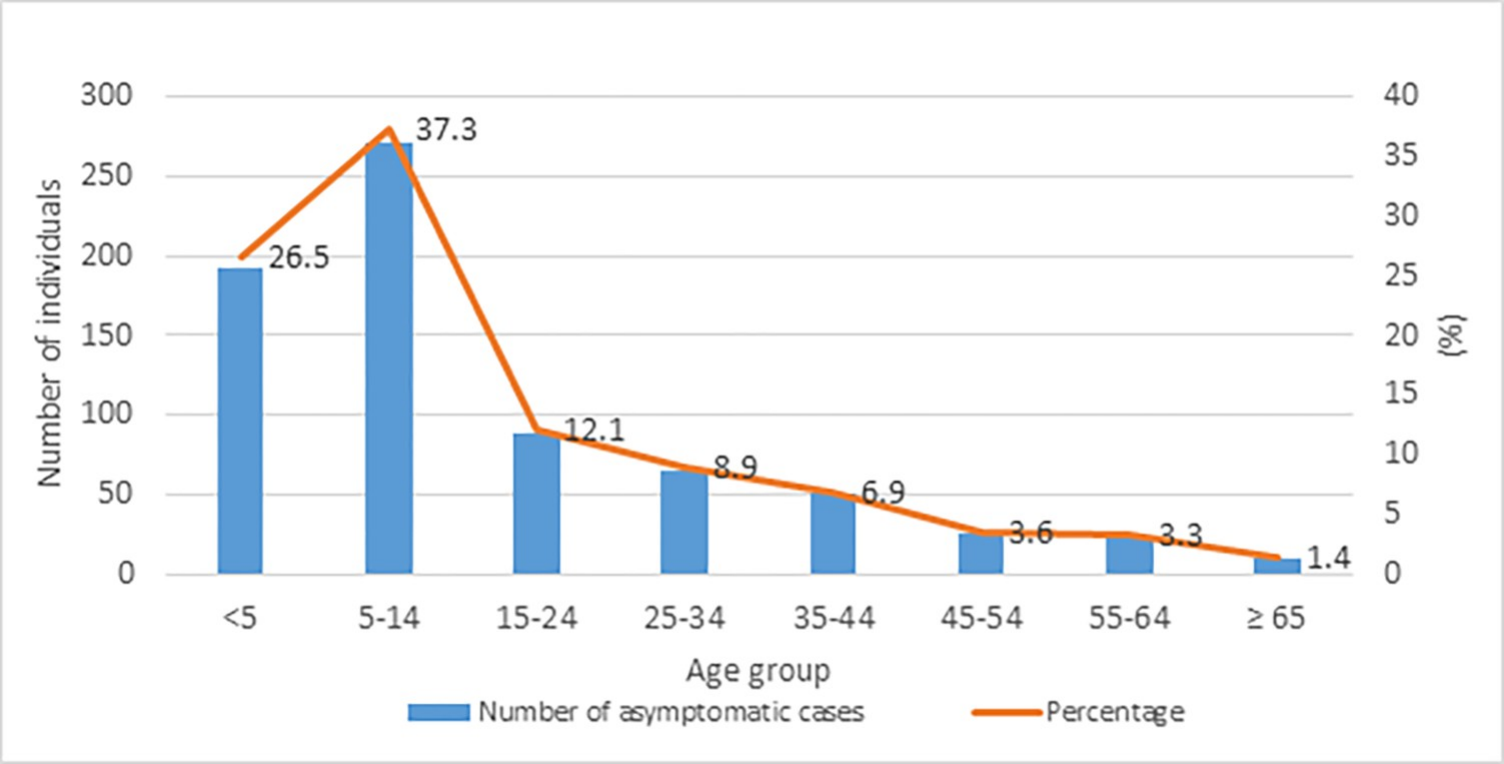
Distribution of asymptomatic carriers in Sabah by age group. Most asymptomatic carriers are children under 15 years of age.

**Fig 7:**
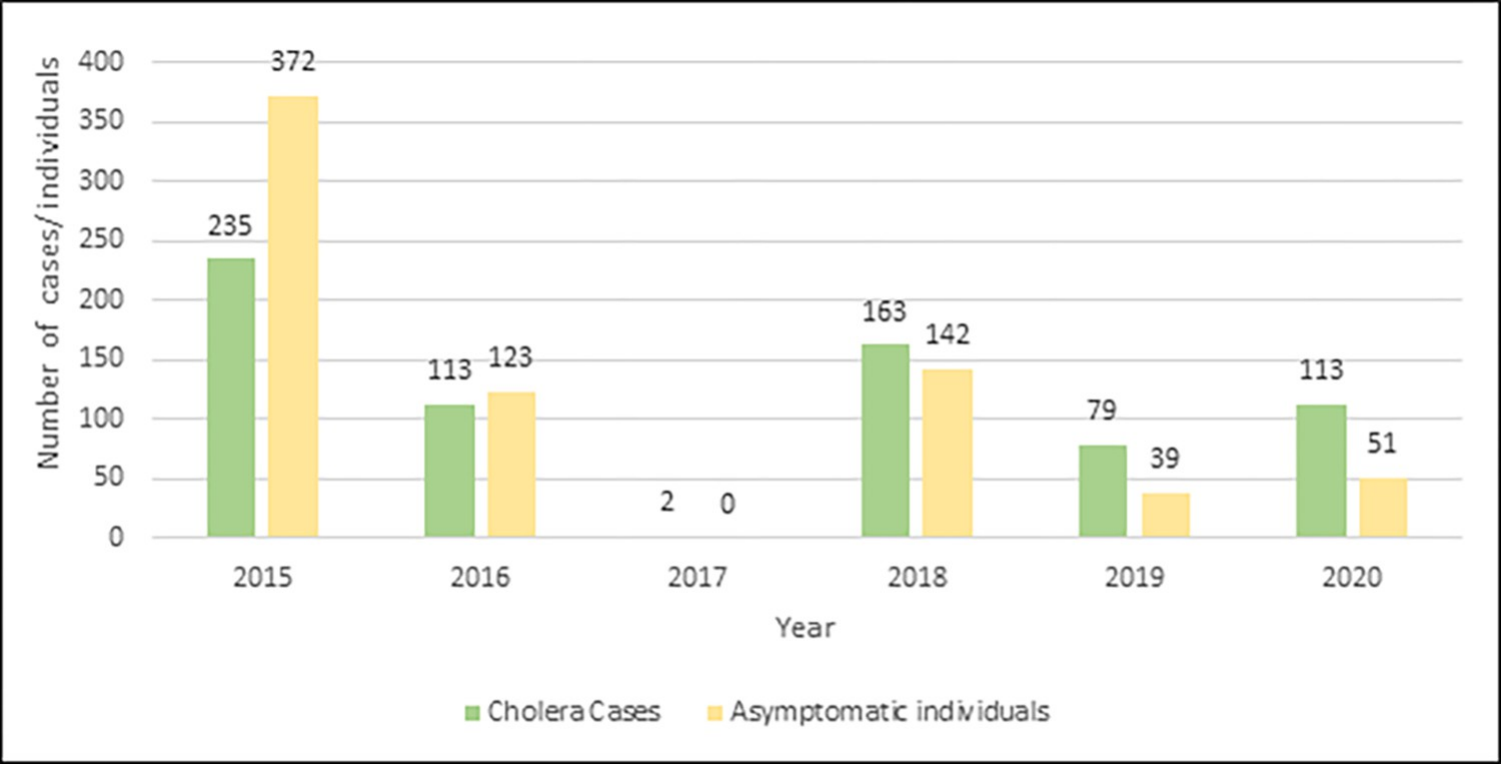
Yearly distribution of cholera cases and asymptomatic carriers in Sabah. Asymptomatic carriers are more than cholera cases in 2015 and 2016 but the reverse is observed in 2018, 2019 and 2020.

Data on asymptomatic cholera were available from 2015 onwards; from 2015 to 2020, a total of 727 asymptomatic cholera cases were detected. The majority of asymptomatic cases were classified as Malaysian (55.4%, 403/727 cases), followed by Filipinos (38.2%, 278/727 cases), Indonesians (1%, 7/727 cases) and 5.4% (39/727 cases) in Sea Gypsies (Table 1). Similar numbers of male and female asymptomatic carriers were detected (358 females and 359 males). Most asymptomatic cholera cases (63.8%, 464/727) were individuals below the age of 15 years old (Fig 6). A comparison between the annual numbers of symptomatic and asymptomatic cholera cases showed higher proportions of asymptomatic cases were detected during 2015 and 2016, whereas from 2017 onwards higher numbers of symptomatic cases were detected (Fig 7).

### Mapping and spatiotemporal clustering

The average population density in cholera-affected villages was 19.45 person/km^2^, lower than the mean population density across Sabah (52.32 person/km^2^). Cases were most frequently reported near coastal areas and at low elevations, with mean (19.45m) and median (9.0 m) above sea level (Table 2). There was marked spatial and temporal heterogeneity in cholera detections across this time period (S1 Video).

**Table 2:** Descriptive statistics for environmental characteristics at locations of cholera incident cases between 2015–2020 (n = 705).

	Cholera cases			
	Mean	Range	Median	IQR
Age (years)	21.7	0–86	15	4–35
Distance from coast (m)	2698.03	3.39–88585.01	655.43	186.22–2742.89
Population density (person/km^2^)	19.45	0.06–72.37	11.1	1.79–33.62
Elevation (m)	21.90	-3.00–1048.00	9.0	3.00–20.00

To evaluate spatial clustering, tau statistics were first calculated within the standard dispersal range for cholera, up to 550m. We found a zone of increased risk of contracting cholera extending until approximately 175m from the primary case, after which the relative risk lowers and stabilises around simulated spatial randomness (S1 Fig). Within 0–5 days after a cholera case being notified at a health facility, those within 0–50m of the primary case had a 15.06-fold increased risk (95% CI: 11.60–19.50) of becoming a cholera case relative to the general population of reported cases in Sabah. At 100–150m distance from the primary cholera case the relative risk continues to be elevated, with 10.76-fold increase in risk (95% CI: 0.00–28.55) relative to the general population within 0–5 days of primary case presentation. However, there are high degrees of uncertainty around tau estimates (illustrated by wide 95% confidence intervals), with relative risk that approximates estimates generated under assumptions of spatial randomness (S1 Fig). This uncertainty is likely a result of village level geolocation creating data sparsity at distances below 1km.

To account for the geographic uncertainty in cholera case data geolocated to village centroids and investigate spatiotemporal linkage between villages, we explored estimating tau statistics for a wider geographic area. Within a 10 km range, we found a clear pattern of spatial risk up to 2 km and within 0–5 days of initial case notification, with elevated risk at shorter distances from the village location of the primary case. At distances of 0–1000m, risk of cholera is 11.41-fold higher than risk anywhere in the general population within 0–5 days of primary case presentation (95% CI: 7.46–16.68). At 1000–2000m, risk of cholera drops to 4.52-fold higher relative to the general population (95% CI: 2.05–7.60), remaining relatively stable until 5km. Above 5km, there appears to be no discernible difference between tau estimates and estimates generated under spatial randomness, indicating that any trends above 5km can be considered noise.

**Fig 8:**
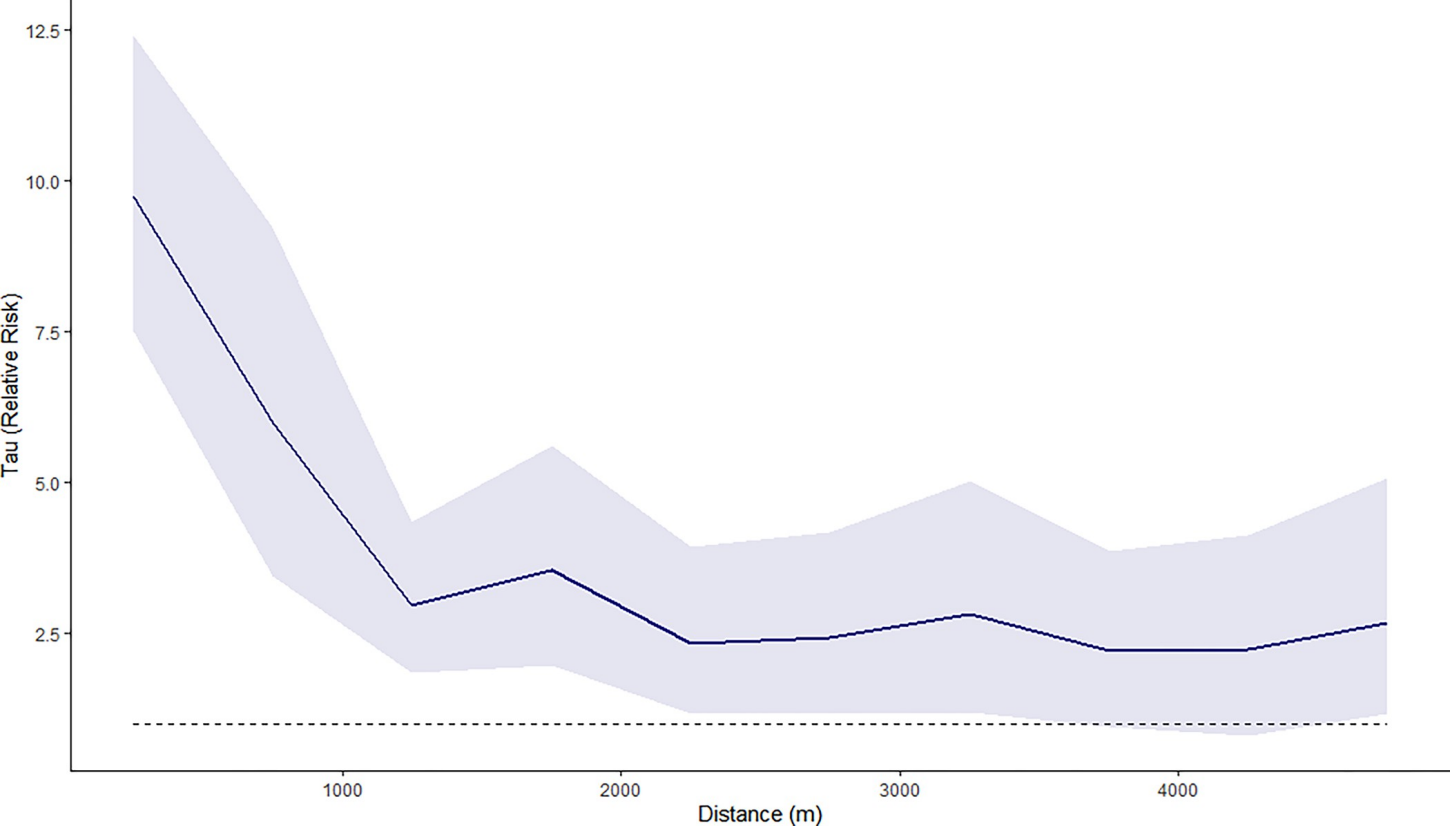
Tau statistic for cholera in Sabah, 2015–2020. Solid line represents estimates of the tau (relative risk) of the next cholera case being within a specified distance to another case within 0–5 days, compared to the risk of the case occurring anywhere in the population within the same time period up to 5km. Blue ribbon represents 95% confidence interval around tau estimates, calculated from 1000 bootstrap replicates. Dashed line represents zero risk (RR = 1). *N = 705* symptomatic cases presenting to health facilities.

Given that trends appear to be significant within a 5-kilometre range, spatiotemporal clustering was then explored within 5km, at narrower bands of 500m. Within 0–5 days of an initial village cholera case, risk of cholera is still notably elevated for all distances up until 5km (5000 meters), with risk estimates remaining above estimates generated under assumptions of spatial randomness. There is evidence of significant clustering within distances of 0–500m, with a 9.74-fold increase in risk of cholera (95% CI: 7.51–12.41) within 0–5 days of initial case presentation to a health facility (Fig 8). At 500-1000m, there is a lesser but still high risk of cholera occurring in the first 5 days, with 6.01-fold higher risk after primary case presentation relative to the general population (3.65–9.07). By 1000–1500 meters, risk of cholera within 0–5 days has reduced to 2.98-fold higher than the population (1.96–5.56), after which tau estimates stabilise at a constant level. Zone of increased risk extends until approximately 3500m (3.5km) from initial case presentation, at which point the 95% CI for tau crosses unity (no elevated risk, RR = 1).

## Discussion

This study documents the distribution of cholera across Sabah and *V*. *cholerae* O1 El Tor as the etiologic agent. Cholera cases were primarily identified in individuals below the age of 15, with no clear associations between cholera risk and gender. Cases were highly clustered in time and space with multiple districts reporting repeat outbreaks, particularly in rural, coastal areas. While further data is needed to fully understand the modes of transmission and importance of asymptomatic reservoirs, this study identifies temporal and spatial characteristics which can be used to prioritize surveillance and control measures.

The average incidence rate in cholera endemic countries is 230/100,000 population; however, the incidence of cholera in Sabah is considerably lower **[1]**. This is likely due to the fact that cholera cases occur in Sabah periodically and are mostly concentrated at underprivileged areas near the coastline. This low incidence may also be due to mandatory notifications of cholera resulting in focused and targeted control and preventive efforts. Additionally, surveillance of cholera has been improved gradually; from 2015 onwards, data on both cholera cases as well as asymptomatic carriers are captured to better reflect the true burden of this disease. Although Malaysian healthcare system is competent however, some cases particularly the asymptomatic carriers may escape the notification.

There remain critical gaps in understanding factors driving the incidence and persistence of cholera in Sabah, with research urgently needed to characterise transmission modes. Other studies have proposed that cholera outbreaks are partially driven by an increase in the hyperinfectious *V*. *cholerae* population and quenched by an increase in virulent phages in infected humans and in environmental reservoirs **[25]**. At least three transmission modes associated with cholera hyperinfectivity have been identified: *V*. *cholerae* shed by humans, biofilm derived *V*. *cholerae*, and the excretion of live *V*. *cholerae* inside expelled food vacuoles by aquatic predatory protozoa **[26]**. Within Sabah, transmission modes remain uncharacterised. While control of outbreaks is possible, this presents challenges for forecasting or preventing future outbreaks. Asymptomatic carriers may contribute to the propagation of cholera outbreaks, as these cases shed *V*. *cholerae* in their stool and may promote the spread of the pathogen. In this study, the highest proportion of asymptomatic carriers were also among children under 15 years old. Conversely, in Kolkata, India, the highest proportion of asymptomatic carriers was reported among adults **[27].** The presence of asymptomatic carriers may be due to acquired immunity in cholera endemic areas **[5, 28].** However, in Sabah, we also found that children represented the highest proportion of clinical cases, in line with global trends **[29].** This may also be due to bias in health-seeking behavior as previous studies have identified that young children with symptoms of diarrhea are more frequently taken for health care treatment compared to adults **[30].** Studies conducted in Nepal and Bangladesh also revealed that children had highest risks of clinical cholera **[31, 32].** In contrast, a 2010 study from Nigeria reported adults were more prone to the cholera, likely due to displacement of the local communities during floods and resulting limited access to safe drinking water **[33].** To understand determinants of symptomatic and asymptomatic cholera infections and the influence of age, characterization of intestinal microbiota is important **[34]** The severity and dissemination of *V*. *cholerae* from host to environment are modulated by auto-inducers, an important bacterial component that triggers expression of numerous virulence factors in *V*. *cholerae* **[35]**.

Although more than half of cholera cases were Malaysian in our study, a substantial proportion of cases were Filipinos and Indonesians and fewer cases reported among the Sea Gypsies. There are frequent cross-border movements of people between Malaysia and the Philippines in the cholera prone districts Semporna, Kunak, Tawau, and Lahad Datu of the east coast of Sabah. These districts correspond to high population movement coupled with issues arising from undocumented immigration, which may contribute to the endemicity and the challenge of controlling cholera outbreaks in Sabah **[17, 36, 37]**. Areas located within the interior of Sabah, including Ranau, Tenom, and Nabawan districts have not reported cholera for the past five years. Compared with coastal districts, the populations in these districts are less mobile. Furthermore, as these districts are located far from the coast, seafood is not regularly consumed by populations within these areas. Previous studies have identified consumption of contaminated street foods from infected food handlers and consumption of undercooked and raw seafood as risk factors for cholera outbreaks in Malaysia **[11]**.

Environmental and meteorological factors are also widely associated with risks of cholera outbreaks. Within Sabah, the highest incidence of cholera was reported in January during the rainy season; however, the majority of cases occurred during the dry seasons from June to September. This is similar to the cholera epidemics in Africa, which are associated with both very dry and very wet conditions **[25]**. Neighboring Indonesia and the Philippines are located on the pacific ring of fire and reported considerable diarrheal diseases outbreaks following natural disaster such as major floods, earthquakes, and typhoons **[38, 39].** Sabah is close neighbor of Indonesian provinces, and in addition it shares maritime borders with the Philippines. The porous sea and land borders coupled with high population movements between Sabah and neighboring Indonesia and the Philippines places the state at constant risk of importation of diarrheal diseases, as well as other communicable infectious diseases **[36, 37]**. Additionally, interannual variations in sea surface temperature might play a role in the epidemiology of cholera in Sabah. In the Bay of Bengal, warm sea surface temperature during El Nino Southern Oscillation events facilitated the growth of environmental reservoir of *V*. *cholerae*, increasing the severity of that year’s epidemic **[40]**. These factors may contribute to interannual and seasonal patterns of cholera outbreaks in Sabah.

Geolocation of the cases and local environmental characteristics suggest that cases mainly occurred in areas with lower population density and at low elevation, consistent with notions that cholera outbreaks occur in rural areas with inadequate sanitation infrastructure, in low-lying coastal areas and in areas vulnerable to contaminated water run-off. Spatiotemporal analysis identified multiple transmission hotspots across Sabah and clear clustering of cases within affected areas, indicating focal transmission occurring between individuals close in space (geographically nearby a primary case) and time (within 5 days of initial case presentation). Clear spatiotemporal clustering of cases within defined areas highlights the need for targeted interventions following identification of index cases to contain outbreaks at their source. Future studies could evaluate patterns of transmission within these affected areas to develop localized control strategies.

Several efforts are in place to contain cholera such as access to WASH (water, sanitation and hygiene) services and health education. Chlorination is done at the source of water of case house for example wells, water containers, and even at rivers by slow release chlorination. All cases and asymptomatic carriers are treated with appropriate antibiotics. Current methods of controlling cholera outbreaks by mass testing and treatment are highly resource intensive, especially in districts with repeated cholera outbreaks such as Kota Kinabalu, Semporna, and Kunak. Therefore, it may be worthwhile to evaluate deploying targeted cholera vaccination programs for high burden areas in Sabah; these measures should be integrated with other strategies including providing safe water, sanitation and hygiene services as well as health promotion and education. Administration of oral cholera vaccines is recommended by the World Health Organization as part of the strategies to end cholera in the global roadmap for 2030 and has been found to be proven effective in areas with cholera outbreaks **[7, 41–43]**. While this study describes cholera risks in Sabah, further investigation of transmission pathways and development of predictive models for areas and populations with high cholera risks is essential to target vaccination campaigns.

This study showed that health promotion should be focused in areas where cross border movements are frequent and sea food is consumed more. Furthermore, prevention strategies, in the form of clean water supply and sanitary toilet, should be prioritized in the rural areas of low elevations coastal regions. These regions might be also selected for targeted vaccination against cholera.

## Conclusion

This retrospective study of case notifications revealed that cholera continues to be a challenge in Sabah and that children are the most vulnerable group affected by this infection. The number of cases varied yearly, mainly affecting the west and east coast districts of Sabah. Most cholera cases were among locals, however, there were also cases identified in people from neighboring countries and Sea Gypsies. Factors such rapid urbanization, climate change, undocumented immigrants and stateless minorities may contribute to cholera outbreaks in Sabah. Cases cluster in space and time, highlighting a need for control measures that contain and prevent disease spread from affected areas. As part of the targeted approach to eliminate cholera in this state, strategies should include vaccination program at selected areas accompanied by public health promotion as well as providing safe water, sanitation and hygiene services.

## Supporting information

S1 Fig Tau statistic for cholera in Sabah, 2015–2020. Solid line represents estimates of the tau (relative risk) of the next cholera case being within a specified distance to another case within 0–5 days, compared to the risk of the case occurring anywhere in the population within the same time period up to 500m. Dashed line represents zero risk (RR = 1). Blue ribbon represents tau estimates simulated under assumptions of spatial randomness, calculated over 1000 permutations. Base maps in were obtained from the UN OCHA Humanitarian Data Exchange platform [Malaysia - Subnational Administrative Boundaries - Humanitarian Data Exchange (humdata.org)], available for use under the following CC BY license conditions [Data Licenses - Humanitarian Data Exchange (humdata.org)].
(TIF)

S1 Table Number of cholera cases, population and incidence by district in Sabah. Data shown here are from 2015 through 2020.
(DOCX)

S2 Table Incidence rate of cholera cases by district in Sabah. Data shown here are from 2015 through 2020.
(DOCX)

S1 Video Cholera detection across time period. There was marked spatial and temporal heterogeneity in cholera detections from 2015 through 2020. Base maps in were obtained from the UN OCHA Humanitarian Data Exchange platform [Malaysia - Subnational Administrative Boundaries - Humanitarian Data Exchange (humdata.org)], available for use under the following CC BY license conditions [Data Licenses - Humanitarian Data Exchange (humdata.org)].
(GIF)
