# Supplementary figures and images for: The incidence, and spatial trends of cholera in Sabah over 15 years: Repeated outbreaks in coastal areas
Source: PLOS Glob Public Health. 2024 Jan 30;4(1):e0002861. doi: 10.1371/journal.pgph.0002861 (PMC10826939; doi:10.1371/journal.pgph.0002861)

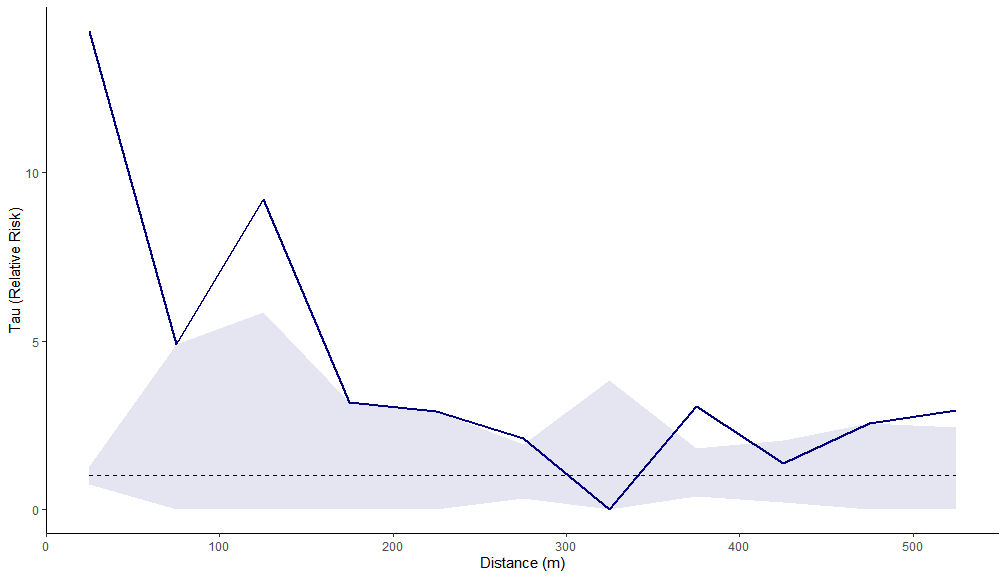

Supplement: S1 Fig — Solid line represents estimates of the tau (relative risk) of the next cholera case being within a specified distance to another case within 0–5 days, compared to the risk of the case occurring anywhere in the population within the same time period up to 500m. Dashed line represents zero risk (RR = 1). Blue ribbon represents tau estimates simulated under assumptions of spatial randomness, calculated over 1000 permutations. Base maps in were obtained from the UN OCHA Humanitarian Data Exchange platform [Malaysia - Subnational Administrative Boundaries - Humanitarian Data Exchange (humdata.org)], available for use under the following CC BY license conditions [Data Licenses - Humanitarian Data Exchange (humdata.org)]. (TIF) [file pgph.0002861.s001.tif]

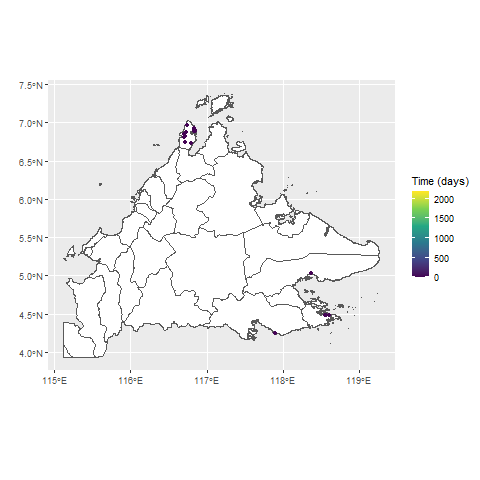

Supplement: S1 Video — There was marked spatial and temporal heterogeneity in cholera detections from 2015 through 2020. Base maps in were obtained from the UN OCHA Humanitarian Data Exchange platform [Malaysia - Subnational Administrative Boundaries - Humanitarian Data Exchange (humdata.org)], available for use under the following CC BY license conditions [Data Licenses - Humanitarian Data Exchange (humdata.org)]. (GIF) [file pgph.0002861.s004.gif]
