# Supplementary material for: The incidence, and spatial trends of cholera in Sabah over 15 years: Repeated outbreaks in coastal areas
Source: PLOS Glob Public Health. 2024 Jan 30;4(1):e0002861. doi: 10.1371/journal.pgph.0002861 (PMC10826939; doi:10.1371/journal.pgph.0002861)
Supplement: S1 Table — Data shown here are from 2015 through 2020. (DOCX) [file pgph.0002861.s002.docx]

| **DISTRICT** | **Number of Case / Population**  **2015** | **Incidence/100000 population**  **2015** | **Number of Case / Population**  **2016** | **Incidence/100000 population**  **2016** | **Number of Case / Population**  **2017** | **Incidence/100000 population**  **2017** | **Number of Case / Population**  **2018** | **Incidence/100000 population**  **2018** | **Number of Case / Population**  **2019** | **Incidence/100000 population**  **2019** | **Number of Case / Population**  **2020** | **Incidence/100000 population**  **2020** |
| --- | --- | --- | --- | --- | --- | --- | --- | --- | --- | --- | --- | --- |
|  |  |  |  |  |  |  |  |  |  |  |  |  |
| Kota Kinabalu | 37 / 437900 | 8.4 | 10 / 437900 | 2.3 | 1 / 509700 | 0.2 | 9 / 515500 | 1.7 | 16 / 563300 | 2.8 | 21 / 572600 | 3.7 |
| Penampang | 6 / 146200 | 4.1 | 1 / 146200 | 0.7 | 0 / 155800 | 0.0 | 0 / 159900 | 0.0 | 0 / 150500 | 0.0 | 2 / 152900 | 1.3 |
| Putatan | 8 / 68700 | 11.6 | 1 / 68700 | 1.5 | 0 / 68800 | 0.0 | 0 / 70600 | 0.0 | 8 / 69900 | 11.4 | 3 / 71500 | 4.3 |
| Papar | 8 / 165200 | 4.8 | 2 / 165200 | 1.2 | 1 / 178500 | 0.6 | 1 / 185100 | 0.5 | 0 / 163700 | 0.0 | 1 / 167300 | 0.6 |
| Tuaran | 6 / 117800 | 5.1 | 1 / 117800 | 0.8 | 0 / 130900 | 0.0 | 3 / 134300 | 2.2 | 5 / 125900 | 4.0 | 3 / 128200 | 2.4 |
| Ranau | 0 / 163800 | 0.0 | 0 / 163800 | 0.0 | 0 / 111100 | 0.0 | 0 / 120300 | 0.0 | 0 / 112100 | 0.0 | 0 / 114000 | 0.0 |
| Kota Belud | 3 / 100200 | 3.0 | 24 / 100200 | 24.0 | 0 / 117200 | 0.0 | 0 / 113700 | 0.0 | 0 / 109900 | 0.0 | 0 / 111800 | 0.0 |
| Kudat | 44 / 85000 | 51.8 | 9 / 85000 | 10.6 | 0 / 89100 | 0.0 | 1 / 89400 | 1.1 | 1 / 100200 | 1.0 | 0 / 101700 | 0.0 |
| Kota Marudu | 2 / 74000 | 2.7 | 0 / 74000 | 0.0 | 0 / 77100 | 0.0 | 0 / 78300 | 0.0 | 0 / 79900 | 0.0 | 0 / 81300 | 0.0 |
| Pitas | 2 / 46000 | 4.3 | 0 / 46000 | 0.0 | 0 / 43700 | 0.0 | 0 / 44300 | 0.0 | 0 / 44700 | 0.0 | 1 / 45400 | 2.2 |
| Sandakan | 11 / 427700 | 2.6 | 20 / 427700 | 4.7 | 0 / 447300 | 0.0 | 6 / 452000 | 1.3 | 0 / 502900 | 0.0 | 0 / 510600 | 0.0 |
| Kinabatangan | 1 / 165800 | 0.6 | 2 / 165800 | 1.2 | 0 / 169100 | 0.0 | 0 / 171600 | 0.0 | 0 / 195400 | 0.0 | 1 / 198100 | 0.5 |
| Tongod | 0 / 42700 | 0.0 | 1 / 42700 | 2.3 | 0 / 41400 | 0.0 | 0 / 42100 | 0.0 | 0 / 43600 | 0.0 | 0 / 44200 | 0.0 |
| Beluran | 55 / 108700 | 50.6 | 2 / 108700 | 1.8 | 0 / 119900 | 0.0 | 0 / 121700 | 0.0 | 0 / 131500 | 0.0 | 0 / 133500 | 0.0 |
| Tawau | 8 / 417500 | 1.9 | 8 / 417500 | 1.9 | 0 / 441500 | 0.0 | 13 / 444900 | 2.9 | 9 / 506700 | 1.8 | 11 / 514000 | 2.2 |
| Semporna | 35 / 141500 | 24.7 | 14 / 141500 | 9.9 | 0 / 155100 | 0.0 | 81 / 157400 | 51.5 | 11 / 169900 | 6.5 | 53 / 172900 | 31.2 |
| Kunak | 2 / 70200 | 2.8 | 14 / 70200 | 19.9 | 0 / 72700 | 0.0 | 9 / 73900 | 12.2 | 13 / 79100 | 16.4 | 11 / 80300 | 14.0 |
| Lahad Datu | 5 / 225400 | 2.2 | 1 / 225400 | 0.4 | 0 / 239700 | 0.0 | 40 / 244100 | 16.4 | 16 / 255200 | 6.3 | 1 / 259200 | 0.4 |
| Keningau | 1 / 194000 | 0.5 | 2 / 194000 | 1.0 | 0 / 197400 | 0.0 | 0 / 200000 | 0.0 | 0 / 215600 | 0.0 | 0 / 219100 | 0.0 |
| Tambunan | 1 / 38300 | 2.6 | 0 / 38300 | 0.0 | 0 / 42400 | 0.0 | 0 / 43300 | 0.0 | 0 / 42600 | 0.0 | 0 / 43400 | 0.0 |
| Tenom | 0 / 61400 | 0.0 | 0 / 61400 | 0.0 | 0 / 67500 | 0.0 | 0 / 69000 | 0.0 | 0 / 67600 | 0.0 | 0 / 68800 | 0.0 |
| Nabawan | 0 / 35600 | 0.0 | 0 / 35600 | 0.0 | 0 / 37900 | 0.0 | 0 / 38800 | 0.0 | 0 / 39100 | 0.0 | 0 / 39800 | 0.0 |
| Beaufort | 0 / 85000 | 0.0 | 0 / 85000 | 0.0 | 0 / 77100 | 0.0 | 0 / 78400 | 0.0 | 0 / 81800 | 0.0 | 4 / 83400 | 4.9 |
| Kuala Penyu | 0 / 23300 | 0.0 | 0 / 23300 | 0.0 | 0 / 25300 | 0.0 | 0 / 26000 | 0.0 | 0 / 24000 | 0.0 | 0 / 24500 | 0.0 |
| Sipitang | 0 / 43400 | 0.0 | 1 / 43400 | 2.3 | 0 / 45900 | 0.0 | 0 / 47200 | 0.0 | 0 / 44300 | 0.0 | 1 / 45100 | 2.3 |
| **TOTAL** | **235 / 3485300** |  | **113 / 3485300** |  | **2 / 3662100** |  | **163 / 3721800** |  | **79 / 3919400** |  | **113 / 3983600** |  |
