# Supplementary material for: The incidence, and spatial trends of cholera in Sabah over 15 years: Repeated outbreaks in coastal areas
Source: PLOS Glob Public Health. 2024 Jan 30;4(1):e0002861. doi: 10.1371/journal.pgph.0002861 (PMC10826939; doi:10.1371/journal.pgph.0002861)
Supplement: S2 Table — Data shown here are from 2015 through 2020. (DOCX) [file pgph.0002861.s003.docx]

| No. | District | 2015 | 2016 | 2017 | 2018 | 2019 | 2020 | Mean (IR) |
| --- | --- | --- | --- | --- | --- | --- | --- | --- |
| 1 | Kota Kinabalu | 8.4 | 2.3 | 0.2 | 1.7 | 2.8 | 3.7 | 3.2 |
| 2 | Penampang | 4.1 | 0.7 | 0.0 | 0.0 | 0.0 | 1.3 | 1.0 |
| 3 | Putatan | 11.6 | 1.5 | 0.0 | 0.0 | 11.4 | 4.3 | 4.8 |
| 4 | Papar | 4.8 | 1.2 | 0.6 | 0.5 | 0.0 | 0.6 | 1.3 |
| 5 | Tuaran | 5.1 | 0.8 | 0.0 | 2.2 | 4.0 | 2.4 | 2.4 |
| 6 | Ranau | 0.0 | 0.0 | 0.0 | 0.0 | 0.0 | 0.0 | 0.0 |
| 7 | Kota Belud | 3.0 | 24.0 | 0.0 | 0.0 | 0.0 | 0.0 | 4.5 |
| 8 | Kudat | 51.8 | 10.6 | 0.0 | 1.1 | 1.0 | 0.0 | 10.7 |
| 9 | Kota Marudu | 2.7 | 0.0 | 0.0 | 0.0 | 0.0 | 0.0 | 0.5 |
| 10 | Pitas | 4.3 | 0.0 | 0.0 | 0.0 | 0.0 | 2.2 | 1.1 |
| 11 | Sandakan | 2.6 | 4.7 | 0.0 | 1.3 | 0.0 | 0.0 | 1.4 |
| 12 | Kinabatangan | 0.6 | 1.2 | 0.0 | 0.0 | 0.0 | 0.5 | 0.4 |
| 13 | Tongod | 0.0 | 2.3 | 0.0 | 0.0 | 0.0 | 0.0 | 0.4 |
| 14 | Beluran | 50.6 | 1.8 | 0.0 | 0.0 | 0.0 | 0.0 | 8.7 |
| 15 | Tawau | 1.9 | 1.9 | 0.0 | 2.9 | 1.8 | 2.2 | 1.8 |
| 16 | Semporna | 24.7 | 9.9 | 0.0 | 51.5 | 6.5 | 31.2 | 20.6 |
| 17 | Kunak | 2.8 | 19.9 | 0.0 | 12.2 | 16.4 | 14.0 | 10.9 |
| 18 | Lahad Datu | 2.2 | 0.4 | 0.0 | 16.4 | 6.3 | 0.4 | 4.3 |
| 19 | Keningau | 0.5 | 1.0 | 0.0 | 0.0 | 0.0 | 0.0 | 0.3 |
| 20 | Tambunan | 2.6 | 0.0 | 0.0 | 0.0 | 0.0 | 0.0 | 0.4 |
| 21 | Tenom | 0.0 | 0.0 | 0.0 | 0.0 | 0.0 | 0.0 | 0.0 |
| 22 | Nabawan | 0.0 | 0.0 | 0.0 | 0.0 | 0.0 | 0.0 | 0.0 |
| 23 | Beaufort | 0.0 | 0.0 | 0.0 | 0.0 | 0.0 | 4.9 | 0.8 |
| 24 | Kuala Penyu | 0.0 | 0.0 | 0.0 | 0.0 | 0.0 | 0.0 | 0.0 |
| 25 | Sipitang | 0.0 | 2.3 | 0.0 | 0.0 | 0.0 | 2.3 | 0.8 |
